# Microstate transition entropy during hyperexcitability in Alzheimer's disease and sleep and its associations with cognitive decline

**DOI:** 10.1002/alz70856_106495

**Published:** 2026-01-08

**Authors:** Sebastian Moguilner, Courtney Berezuk, Alex C Bender, Kyle R Pellerin, Stephen N Gomperts, Sydney Cash, Rani A Sarkis, Alice D Lam

**Affiliations:** ^1^ Massachusetts General Hospital, Boston, MA, USA; ^2^ LC Campbell Cognitive Neurology Research Unit, Toronto, MA, USA; ^3^ Harvard Medical School, Boston, MA, USA

## Abstract

**Background:**

Sleep disturbances affect nearly 60% of people with Alzheimer's disease (AD) at clinical and preclinical stages. Hyperexcitability in AD also arises during sleep and can lead to epileptiform activity and seizures that impact memory consolidation during N2 sleep. Understanding the millisecond‐scale dynamic activation patterns in EEG topography (i.e., microstates) can provide neurophysiological insights. However, no studies have examined microstate dynamics during sleep and hyperexcitability in AD and its relationship with cognitive decline.

**Method:**

Employing ambulatory scalp EEG recordings, we examined 33 cognitively normal healthy older adult controls (HC), 36 individuals with early clinical stages of AD without history or risk factors for epilepsy (AD‐NoEp), and 14 individuals with early clinical stages of AD who developed epilepsy related to Alzheimer's (AD‐Ep). The analysis involved extracting microstate patterns across sleep‐wake stages (Awake, N2, and REM) and analyzing its entropy to assess the randomness of dynamic transitions between microstates in each group. We also examined cross‐sectional associations and longitudinal interactions with MoCA scores to evaluate relationships between entropy and cognitive decline.

**Result:**

We found specific microstate entropy differences in Alzheimer's across sleep stages. The AD‐NoEp group showed higher entropy compared to HC in both the awake state (*p* = 0.031, Cohen's d = 0.552) and in REM sleep (*p* < 0.001, Cohen's d = 0.94). The AD‐Ep group had reduced entropy in REM compared to AD‐NoEp (*p* = 0.01, Cohen's d = 0.972), with entropy values closer to HC. Cross‐sectional analysis did not demonstrate any significant association between MoCA score and entropy measures across sleep states. However, longitudinal analysis demonstrated a significant interaction effect of entropy during N2 sleep and time on longitudinal MoCA score (*p* = 0.044), where increased entropy during N2 sleep was associated with a faster rate of decline on MoCA.

**Conclusion:**

We found specific changes in microstate transition entropy in AD with and without epilepsy and across the sleep‐wake cycle. Increased entropy in N2 sleep, a stage associated with memory consolidation, was predictive of cognitive decline. Our results offer new insights into understanding brain‐altered dynamics during sleep in AD and open venues to develop new prognostic tools for cognitive decline.

